# Complete genome sequence of a human bacteremia isolate of *Kalamiella piersonii*

**DOI:** 10.1128/MRA.00293-23

**Published:** 2023-08-31

**Authors:** Mondraya Howard, Joel J. Maki, Sara Connelly, Dwight J. Hardy, Andrew Cameron

**Affiliations:** 1 Department of Pathology and Laboratory Medicine, University of Rochester Medical Center, Rochester, New York, USA; 2 Department of Microbiology and Immunology, University of Rochester Medical Center, Rochester, New York, USA; University of Maryland School of Medicine, Baltimore, Maryland, USA

**Keywords:** *Kalamiella*, *Pantoea*, Enterobacteriaceae, blood culture, bloodstream infections

## Abstract

The complete genome of *Kalamiella piersonii* clinical isolate URMC-2103A041 from human bacteremia was determined using the hybrid assembly of short- and long-read sequencing chemistry. The genome contains a 3.93 Mb chromosome, three circular plasmids, and one linear plasmid.

## ANNOUNCEMENT

*Kalamiella piersonii* is a Gram-negative Enterobacterales in the Erwiniaceae family. First described from the International Space Station environment, a recent phylogenomic analysis of Erwiniaceae has proposed reclassification as *Pantoea piersonii* comb. nov (1, 2).

From humans, *K. piersonii* has been isolated from saliva and urine, the latter involving a patient with kidney stone disease, suggesting potential roles in human infection (3, 4). URMC-2103A041 was recovered from a patient whose bacteremia was previously described (5). The organism was detected after 9 hours of incubation at 37°C using a BACT/ALERT SA blood culture bottle on a Virtuo automated blood culture instrument (bioMérieux). Colony growth was isolated on tryptic soy agar with 5% sheep blood agar plates (BAP) after 24 hours at 35°C.

MALDI-TOF (MALDI Biotyper, Bruker) identified the organism as *Pantoea septica* [log score of 1.8 (low confidence)]. Biochemical identification (API 20E, bioMérieux) produced *Pantoea* spp. (98.2% identification). The BD Phoenix NID panel identification was *Shigella flexneri*. Identification was resolved by 16S rRNA V1-V3 sequencing (MicroSeq 500 16S rDNA Sequening Kit) (6) showing 100% identity with *K. piersonii* (Pathogenomix database; Genbank: OR250797.1). Susceptibility was determined using zone diameter breakpoints for Enterobacterales (CLSI M100, 30th Ed.) (7).

**Fig 1:**
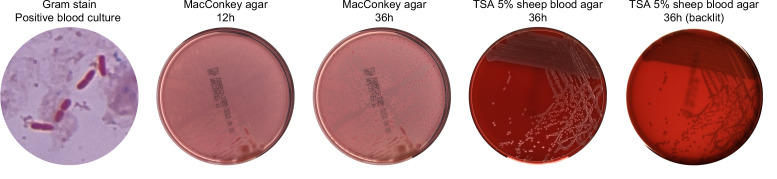
Gram stain and colonial appearance of *K. piersonii* URMC-2103A041.

The bacilli were non-hemolytic and a late lactose fermenter (Fig. 1A). Like other *Pantoea* spp., URMC-2103A041 was Voges-Proskauer- and ONPG-positive, fermented mannitol, arabinose, and rhamnose, but was arginine dihydrolase positive (8, 9).

For DNA extraction of both short- and long-read sequencing library preparation, colonies were purified on a BAP, then inoculated into tryptic soy broth, and incubated for 18 hours (30°C shaking at 200 rpm). DNA was extracted following the protocol for “Pretreatment for Gram-Negative Bacteria” (DNeasy Blood and Tissue kit; Qiagen). DNA shearing and size selection were not performed. DNA was quantified with the QuantiFluor dsDNA system (Promega).

For short reads, paired-end sequencing following library preparation (DNA Prep Kit, Illumina) was performed on a MiSeq instrument (MiSeq Reagent v2 500-cycle kit, Illumina). Unless otherwise specified, sequences were analyzed with tools available on the Galaxy server (v22.05, usegalaxy.eu), using default parameters (10). Reads were trimmed with Trimmomatic v0.36 (11) and quality checked with FastQC v0.11.5 (12) to generate 645,637 paired-end reads (average read length: 224 bp).

For long-read sequencing, 40 ng DNA was used to prepare libraries with the Rapid Barcoding Sequencing Kit [Oxford Nanopore Technologies (ONT), SQK-RBK004]. Long-read libraries were sequenced (MinION flow cell, FLO-MIN106), and base calling was performed with Guppy v6.1.5 (MinKNOW v22.05.5). Filtering (quality score of >8) generated 93,023 reads (read N_50_: 11,125 bp) for hybrid assembly (Unicycler v0.5.0, with rotation to default start gene) and polishing (Quast v5.2.0) (13, 14).

The hybrid assembly genome was 4,990,348 bp with 57.02% GC content; it consisted of one circular chromosome (3.93 Mb, GenBank: CP115895.1), three circular plasmids (527.8 kb, CP115896.1; 277.8 kb, CP115897.1; and 174.2 kb, CP115898.1), and one linear plasmid (81.1 kb, CP115899.1). Annotation was added by the NCBI Prokaryotic Genome Annotation Pipeline (15 - 17).

## Data Availability

The genome sequence and raw sequencing reads for URMC-2103A041 were deposited under BioProject accession number PRJNA916888 (BioSample accession number SAMN32512754) and SRA accession numbers SRX18896138 and SRX18895383. The partial 16S sequence was deposited under Genbank accession number OR250797.1.
